# 3,3′-[1,2-Phenyl­enebis(methyl­ene)]bis­(1-heptyl­benzimidazolium) dibromide monohydrate

**DOI:** 10.1107/S1600536811023476

**Published:** 2011-06-25

**Authors:** Rosenani A. Haque, Muhammad Adnan Iqbal, Madhukar Hemamalini, Hoong-Kun Fun

**Affiliations:** a School of Chemical Sciences, Universiti Sains Malaysia, 11800 USM, Penang, Malaysia; b X-ray Crystallography Unit, School of Physics, Universiti Sains Malaysia, 11800 USM, Penang, Malaysia

## Abstract

In the title salt, C_36_H_48_N_4_^2+^·2Br^−^·H_2_O, the central benzene ring makes dihedral angles of 84.77 (9) and 69.92 (7)° with the adjacent imidazole rings. In the crystal, one of the heptyl groups is disordered over two sets of sites with an occupancy ratio of 0.474 (5):0.526 (5). In the crystal, the cations, anions and water mol­ecules are connected *via* inter­molecular O—H⋯Br, C—H⋯Br and C—H⋯O hydrogen bonds, forming a three-dimensional network.

## Related literature

For details and applications of *N*-heterocyclic carbenes (NHCs), see: Winkelmann & Navarro (2010 ▶); Kascatan-Nebioglu *et al.* (2007 ▶); Teyssot *et al.* (2009 ▶); Herrmann *et al.* (1995 ▶); Choi *et al.* (2001 ▶); Kumar & Kumar (2009 ▶). For the stability of the temperature controller used in the data collection, see: Cosier & Glazer (1986 ▶).
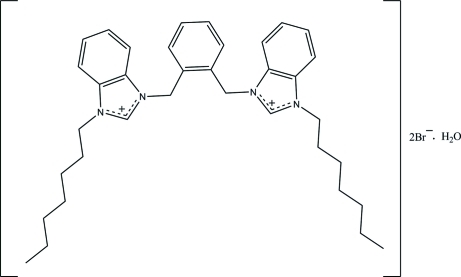


## Experimental

### 

#### Crystal data

C_36_H_48_N_4_^2+^·2Br^−^·H_2_O
*M* *_r_* = 714.62
Triclinic, 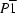

*a* = 8.8494 (1) Å
*b* = 14.7170 (3) Å
*c* = 16.0838 (2) Å
α = 115.705 (1)°
β = 105.380 (1)°
γ = 91.946 (1)°
*V* = 1792.83 (5) Å^3^
*Z* = 2
Mo *K*α radiation
μ = 2.29 mm^−1^
*T* = 100 K
0.39 × 0.18 × 0.16 mm

#### Data collection

Bruker SMART APEXII CCD area-detector diffractometer
Absorption correction: multi-scan (*SADABS*; Bruker, 2009 ▶) *T*_min_ = 0.469, *T*_max_ = 0.715
50860 measured reflections
12945 independent reflections
10091 reflections with *I* > 2σ(*I*)
*R*_int_ = 0.027

#### Refinement

*R*[*F* ^2^ > 2σ(*F* ^2^)] = 0.032
*wR*(*F* ^2^) = 0.077
*S* = 1.02
12945 reflections
442 parameters
9 restraints
H atoms treated by a mixture of independent and constrained refinement
Δρ_max_ = 0.84 e Å^−3^
Δρ_min_ = −0.75 e Å^−3^

### 

Data collection: *APEX2* (Bruker, 2009 ▶); cell refinement: *SAINT* (Bruker, 2009 ▶); data reduction: *SAINT*; program(s) used to solve structure: *SHELXTL* (Sheldrick, 2008 ▶); program(s) used to refine structure: *SHELXTL*; molecular graphics: *SHELXTL*; software used to prepare material for publication: *SHELXTL* and *PLATON* (Spek, 2009 ▶).

## Supplementary Material

Crystal structure: contains datablock(s) global, I. DOI: 10.1107/S1600536811023476/is2732sup1.cif

Structure factors: contains datablock(s) I. DOI: 10.1107/S1600536811023476/is2732Isup2.hkl

Additional supplementary materials:  crystallographic information; 3D view; checkCIF report

## Figures and Tables

**Table 1:** Hydrogen-bond geometry (Å, °)

*D*—H⋯*A*	*D*—H	H⋯*A*	*D*⋯*A*	*D*—H⋯*A*
O1*W*—H1*W*1⋯Br1	0.84 (3)	2.50 (3)	3.3271 (17)	169 (2)
O1*W*—H2*W*1⋯Br2	0.79 (3)	2.54 (3)	3.3280 (14)	177 (3)
C1—H1*A*⋯Br1^i^	0.95	2.80	3.6093 (15)	144
C3—H3*A*⋯Br2^ii^	0.95	2.92	3.7866 (16)	153
C5—H5*A*⋯Br2^iii^	0.95	2.89	3.8162 (17)	167
C8—H8*A*⋯Br2^iv^	0.99	2.93	3.9117 (16)	172
C15—H15*A*⋯Br2^iv^	0.99	2.72	3.6809 (19)	165
C15—H15*B*⋯Br1^iv^	0.99	2.80	3.7842 (15)	170
C18—H18*A*⋯O1*W*^v^	0.95	2.46	3.187 (2)	133
C20—H20*A*⋯Br2	0.95	2.76	3.6602 (16)	158
C22—H22*A*⋯Br1^i^	0.95	2.70	3.5577 (15)	150
C23—H23*A*⋯Br2^i^	0.99	2.89	3.7836 (14)	151
C23—H23*B*⋯Br2^ii^	0.99	2.81	3.7285 (17)	154

Symmetry codes: (i) 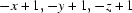; (ii) 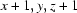; (iii) 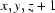; (iv) 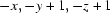; (v) 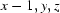.

**Fig. 1.**
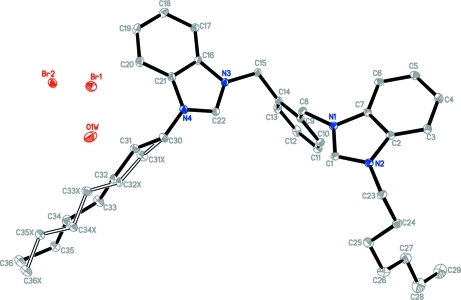
The asymmetric unit of the title compound, showing 30% probability displacement ellipsoids and the atom-numbering scheme. H atoms have been omitted for clarity.

**Fig. 2.**
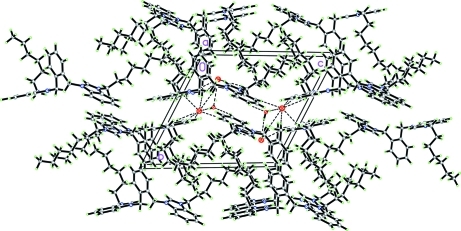
The crystal packing of the title compound, showing hydrogen-bonded (dashed lines) network.

## supplementary crystallographic information

### Comment

*N*-Heterocyclic Carbenes (NHCs) is a versatile class of ligands, which have widespread applications in organometallic chemistry (Winkelmann & Navarro, 2010).  Metal complexes of NHCs have proven to be potential antimicrobial (Kascatan-Nebioglu *et al.*, 2007) and anticancer (Teyssot *et al.*, 2009) agents. Notably, NHCs also exhibit excellent catalytical activity for Heck and Suzuki coupling reactions (Herrmann *et al.*, 1995) and Metathesis Cross-Coupling reactions (Choi *et al.*, 2001). Benzimidazole-based NHCs of similar structures and their metal complexes are now known to be effective catalysts for the cross coupling reactions of different alcohols and ratiometric sensing (Kumar & Kumar, 2009).

The asymmetric unit of the title compound, (Fig. 1), consists of a 3,3'-[1,2-phenylenebis(methylene)]bis(1-heptylbenzimidazolium) cation, two bromine anions and one water molecule. One of the heptyl group is disordered over two sets of sites, with an occupancy ratio of 0.474 (5):0.526 (5). The central benzene (C9–C14) ring makes dihedral angles of 84.77 (9) and  69.92 (7)° with the adjacent imidazole (N1/N2/C1/C2/C7) and (N3/N4/C16/C21/C22) rings, respectively.

In the crystal structure (Fig. 2), the cations, anions and water molecules are linked together *via* intermolecular O1W—H1W1···Br1, O1W—H2W1···Br2, C1—H1A···Br1, C3—H3A···Br2, C5—H5A···Br2, C8—H8A···Br2, C15—H15A···Br2, C15—H15B···Br1, C18—H18A···O1W, C20—H20A···Br2, C22—H22A···Br1, C23—H23A···Br2 and C23—H23B···Br2 (Table 1) hydrogen bonds, forming a three-dimensional network.

### Experimental

A mixture of benzimidazole (2.36 g, 20 mmol) and finely ground potassium hydroxide (2.36 g, 30 mmol) in 30 ml of DMSO was stirred at room temperature (27–28 °C) for 30 minutes. 1-bromoheptane (3.14 ml, 20 mmol) was added drop-wise in this consistently stirred mixture with further stirring for 2 h at the same temperature, poured into water (300 ml) and was extracted by chloroform  (5 × 20 ml). The extract was dried by magnesium sulphate and evaporated under reduced pressure to afford *N*-heptylbenzimidazole (1) as a thick yellowish fluid (3.87 g, 89.6%). Furthermore, a mixture of 1 (2.16 g, 10 mmol) and 1,2-bis(bromomethyl)benzene (1.32 g, 5 mmol) in dioxane (30 ml) was refluxed at 90 °C for 12 h. Desired compound (2.2Br) appeared as beige-colored precipitates in dark brown solution. The mixture was filtered and precipitates were washed by fresh dioxane (3 × 5 ml), dried at room temperature for 24 h, and soft lumps so obtained were ground to fine powder (1.72 g, 49.4%). Hot (saturated) solution of 2.2Br in deuterated DMSO (0.5 ml) was cooled to room temperature in NMR tube overnight to get single (prismatic) crystals suitable for X-ray diffraction study.

### Refinement

Atoms H1W1 and H1W2 were located in a difference Fourier map and refined freely [O—H = 0.79 (3)–0.84 (2) Å]. The remaining H atoms were positioned geometrically (C—H = 0.95–0.99 Å) and were refined using a riding model, with *U*_iso_(H) = 1.2 or 1.5*U*_eq_(C). A rotating group model was applied to the methyl groups. One of the heptyl group is disordered over two sets of sites, with an occupancy ratio of 0.474 (5):0.526 (5). SAME restraints were applied in the refinement of the disordered components. In addition, the thermal ellipsoids of C32/C32X and C28/C29 were restrained to be equal.

### Crystal data

**Table:**

C_36_H_48_N_4_^2+^·2Br^−^·H_2_O	*Z* = 2
*M**_r_* = 714.62	*F*(000) = 744
Triclinic, *P*1	*D*_x_ = 1.324 Mg m^−^^3^
Hall symbol: -P 1	Mo *K*α radiation, λ = 0.71073 Å
*a* = 8.8494 (1) Å	Cell parameters from 9922 reflections
*b* = 14.7170 (3) Å	θ = 2.6–33.7°
*c* = 16.0838 (2) Å	µ = 2.29 mm^−^^1^
α = 115.705 (1)°	*T* = 100 K
β = 105.380 (1)°	Block, colourless
γ = 91.946 (1)°	0.39 × 0.18 × 0.16 mm
*V* = 1792.83 (5) Å^3^	

### Data collection

**Table:**

Bruker SMART APEXII CCD area-detector diffractometer	12945 independent reflections
Radiation source: fine-focus sealed tube	10091 reflections with *I* > 2σ(*I*)
graphite	*R*_int_ = 0.027
φ and ω scans	θ_max_ = 32.5°, θ_min_ = 1.5°
Absorption correction: multi-scan (*SADABS*; Bruker, 2009)	*h* = −13→13
*T*_min_ = 0.469, *T*_max_ = 0.715	*k* = −22→22
50860 measured reflections	*l* = −24→24

### Refinement

**Table:**

Refinement on *F*^2^	Primary atom site location: structure-invariant direct methods
Least-squares matrix: full	Secondary atom site location: difference Fourier map
*R*[*F*^2^ > 2σ(*F*^2^)] = 0.032	Hydrogen site location: inferred from neighbouring sites
*wR*(*F*^2^) = 0.077	H atoms treated by a mixture of independent and constrained refinement
*S* = 1.02	*w* = 1/[σ^2^(*F*_o_^2^) + (0.0359*P*)^2^ + 0.6181*P*] where *P* = (*F*_o_^2^ + 2*F*_c_^2^)/3
12945 reflections	(Δ/σ)_max_ = 0.001
442 parameters	Δρ_max_ = 0.84 e Å^−^^3^
9 restraints	Δρ_min_ = −0.75 e Å^−^^3^

### Special details

**Table:**

Experimental. The crystal was placed in the cold stream of an Oxford Cryosystems Cobra open-flow nitrogen cryostat (Cosier & Glazer, 1986) operating at 100.0 (1) K.
Geometry. All s.u.'s (except the s.u. in the dihedral angle between two l.s. planes) are estimated using the full covariance matrix. The cell s.u.'s are taken into account individually in the estimation of s.u.'s in distances, angles and torsion angles; correlations between s.u.'s in cell parameters are only used when they are defined by crystal symmetry. An approximate (isotropic) treatment of cell s.u.'s is used for estimating s.u.'s involving l.s. planes.
Refinement. Refinement of F^2^ against ALL reflections. The weighted R-factor wR and goodness of fit S are based on F^2^, conventional R-factors R are based on F, with F set to zero for negative F^2^. The threshold expression of F^2^ > 2σ(F^2^) is used only for calculating R-factors(gt) etc. and is not relevant to the choice of reflections for refinement. R-factors based on F^2^ are statistically about twice as large as those based on F, and R- factors based on ALL data will be even larger.

### Fractional atomic coordinates and isotropic or equivalent isotropic displacement parameters (Å^2^)

**Table:**

	*x*	*y*	*z*	*U*_iso_*/*U*_eq_	Occ. (<1)
Br1	0.365462 (16)	0.228743 (11)	0.219134 (10)	0.02173 (4)	
Br2	0.031239 (16)	0.512483 (11)	0.193441 (10)	0.02124 (4)	
N1	0.48319 (13)	0.66074 (9)	0.94225 (8)	0.0168 (2)	
N2	0.72144 (13)	0.65150 (9)	1.01988 (8)	0.0172 (2)	
N3	0.07774 (13)	0.69630 (9)	0.68277 (8)	0.0178 (2)	
N4	0.19471 (14)	0.67552 (10)	0.57302 (9)	0.0198 (2)	
C1	0.63655 (16)	0.66612 (10)	0.94634 (10)	0.0171 (2)	
H1A	0.6787	0.6786	0.9030	0.021*	
C2	0.61934 (16)	0.63548 (10)	1.06701 (10)	0.0167 (2)	
C3	0.64670 (17)	0.61359 (11)	1.14536 (10)	0.0193 (3)	
H3A	0.7500	0.6096	1.1792	0.023*	
C4	0.51353 (18)	0.59803 (11)	1.17085 (10)	0.0207 (3)	
H4A	0.5262	0.5831	1.2239	0.025*	
C5	0.36033 (18)	0.60365 (11)	1.12058 (11)	0.0215 (3)	
H5A	0.2727	0.5921	1.1405	0.026*	
C6	0.33375 (16)	0.62550 (11)	1.04280 (10)	0.0193 (3)	
H6A	0.2306	0.6294	1.0088	0.023*	
C7	0.46771 (16)	0.64135 (10)	1.01748 (10)	0.0166 (2)	
C8	0.35503 (16)	0.67445 (11)	0.87201 (10)	0.0187 (3)	
H8A	0.2604	0.6221	0.8496	0.022*	
H8B	0.3889	0.6635	0.8149	0.022*	
C9	0.30936 (16)	0.77994 (11)	0.91357 (10)	0.0177 (3)	
C10	0.40829 (17)	0.86003 (11)	0.99982 (11)	0.0220 (3)	
H10A	0.5053	0.8482	1.0332	0.026*	
C11	0.36713 (19)	0.95694 (12)	1.03770 (11)	0.0252 (3)	
H11A	0.4355	1.0105	1.0967	0.030*	
C12	0.2263 (2)	0.97520 (12)	0.98931 (11)	0.0266 (3)	
H12A	0.1980	1.0413	1.0148	0.032*	
C13	0.12706 (19)	0.89630 (13)	0.90350 (11)	0.0253 (3)	
H13A	0.0305	0.9089	0.8705	0.030*	
C14	0.16660 (16)	0.79853 (11)	0.86476 (10)	0.0193 (3)	
C15	0.04692 (16)	0.71575 (12)	0.77466 (10)	0.0204 (3)	
H15A	0.0452	0.6514	0.7803	0.025*	
H15B	−0.0599	0.7348	0.7715	0.025*	
C16	−0.03893 (16)	0.64533 (11)	0.59265 (10)	0.0183 (3)	
C17	−0.20136 (17)	0.61256 (12)	0.56824 (11)	0.0215 (3)	
H17A	−0.2515	0.6215	0.6160	0.026*	
C18	−0.28532 (18)	0.56616 (12)	0.46997 (11)	0.0250 (3)	
H18A	−0.3966	0.5430	0.4497	0.030*	
C19	−0.21027 (19)	0.55241 (12)	0.39959 (11)	0.0261 (3)	
H19A	−0.2724	0.5198	0.3330	0.031*	
C20	−0.04885 (19)	0.58480 (12)	0.42390 (11)	0.0246 (3)	
H20A	0.0016	0.5754	0.3761	0.029*	
C21	0.03504 (17)	0.63207 (11)	0.52276 (10)	0.0197 (3)	
C22	0.21526 (16)	0.71327 (11)	0.66812 (10)	0.0190 (3)	
H22A	0.3135	0.7472	0.7180	0.023*	
C23	0.89380 (16)	0.64783 (11)	1.04495 (10)	0.0199 (3)	
H23A	0.9285	0.6320	0.9872	0.024*	
H23B	0.9132	0.5921	1.0632	0.024*	
C24	0.99196 (17)	0.74804 (11)	1.12807 (11)	0.0217 (3)	
H24A	1.1005	0.7363	1.1530	0.026*	
H24B	0.9441	0.7700	1.1814	0.026*	
C25	1.0042 (2)	0.83405 (12)	1.10038 (12)	0.0279 (3)	
H25A	0.8967	0.8498	1.0803	0.033*	
H25B	1.0449	0.8106	1.0440	0.033*	
C26	1.1138 (2)	0.93188 (13)	1.18354 (13)	0.0312 (4)	
H26A	1.2184	0.9146	1.2074	0.037*	
H26B	1.1310	0.9804	1.1580	0.037*	
C27	1.0495 (2)	0.98436 (13)	1.26812 (13)	0.0318 (4)	
H27A	1.0368	0.9369	1.2954	0.038*	
H27B	0.9428	0.9987	1.2436	0.038*	
C28	1.1542 (3)	1.08388 (15)	1.34891 (15)	0.0474 (4)	
H28A	1.1636	1.1328	1.3227	0.057*	
H28B	1.2621	1.0704	1.3724	0.057*	
C29	1.0880 (3)	1.13208 (15)	1.43403 (15)	0.0474 (4)	
H29A	1.1598	1.1953	1.4847	0.071*	
H29B	1.0790	1.0841	1.4605	0.071*	
H29C	0.9827	1.1478	1.4116	0.071*	
C30	0.31844 (18)	0.68129 (12)	0.52875 (11)	0.0237 (3)	
H30A	0.3231	0.6112	0.4817	0.028*	0.474 (5)
H30B	0.4229	0.7092	0.5801	0.028*	0.474 (5)
H30C	0.3035	0.6192	0.4701	0.028*	0.526 (5)
H30D	0.4214	0.6897	0.5729	0.028*	0.526 (5)
C31	0.2912 (8)	0.7454 (5)	0.4784 (4)	0.0235 (11)	0.474 (5)
H31A	0.2774	0.8131	0.5252	0.028*	0.474 (5)
H31D	0.1887	0.7142	0.4256	0.028*	0.474 (5)
C32	0.4121 (14)	0.7637 (9)	0.4352 (8)	0.0239 (12)	0.474 (5)
H32A	0.3838	0.7084	0.3677	0.029*	0.474 (5)
H32D	0.5159	0.7554	0.4712	0.029*	0.474 (5)
C33	0.4379 (5)	0.8674 (3)	0.4321 (3)	0.0283 (9)	0.474 (5)
H33A	0.3348	0.8910	0.4194	0.034*	0.474 (5)
H33B	0.5095	0.9198	0.4956	0.034*	0.474 (5)
C34	0.5109 (7)	0.8542 (6)	0.3522 (5)	0.0284 (13)	0.474 (5)
H34A	0.6021	0.8179	0.3584	0.034*	0.474 (5)
H34B	0.4308	0.8102	0.2884	0.034*	0.474 (5)
C35	0.5674 (5)	0.9528 (3)	0.3533 (3)	0.0302 (9)	0.474 (5)
H35A	0.4821	0.9949	0.3581	0.036*	0.474 (5)
H35B	0.6609	0.9917	0.4114	0.036*	0.474 (5)
C36	0.6121 (7)	0.9350 (6)	0.2638 (4)	0.0435 (14)	0.474 (5)
H36A	0.6544	1.0007	0.2699	0.065*	0.474 (5)
H36B	0.5177	0.9018	0.2064	0.065*	0.474 (5)
H36C	0.6932	0.8909	0.2571	0.065*	0.474 (5)
C31X	0.3150 (8)	0.7765 (4)	0.5075 (4)	0.0293 (11)	0.526 (5)
H31B	0.2075	0.7748	0.4674	0.035*	0.526 (5)
H31C	0.3463	0.8414	0.5687	0.035*	0.526 (5)
C32X	0.4359 (12)	0.7661 (8)	0.4522 (7)	0.0239 (12)	0.526 (5)
H32B	0.4350	0.6924	0.4124	0.029*	0.526 (5)
H32C	0.5435	0.7961	0.5000	0.029*	0.526 (5)
C33X	0.4057 (4)	0.8177 (3)	0.3857 (3)	0.0265 (8)	0.526 (5)
H33C	0.3278	0.7700	0.3223	0.032*	0.526 (5)
H33D	0.3582	0.8792	0.4150	0.032*	0.526 (5)
C34X	0.5560 (6)	0.8489 (5)	0.3687 (5)	0.0227 (10)	0.526 (5)
H34C	0.6078	0.7883	0.3439	0.027*	0.526 (5)
H34D	0.6308	0.9005	0.4314	0.027*	0.526 (5)
C35X	0.5249 (4)	0.8934 (3)	0.2968 (3)	0.0292 (8)	0.526 (5)
H35C	0.4514	0.8415	0.2337	0.035*	0.526 (5)
H35D	0.4720	0.9535	0.3210	0.035*	0.526 (5)
C36X	0.6762 (6)	0.9258 (4)	0.2811 (4)	0.0358 (10)	0.526 (5)
H36D	0.6505	0.9590	0.2391	0.054*	0.526 (5)
H36E	0.7231	0.8653	0.2503	0.054*	0.526 (5)
H36F	0.7524	0.9738	0.3439	0.054*	0.526 (5)
O1W	0.39216 (15)	0.47948 (11)	0.29323 (9)	0.0324 (3)	
H1W1	0.380 (3)	0.4177 (19)	0.2809 (17)	0.050 (7)*	
H2W1	0.307 (3)	0.4896 (18)	0.2719 (17)	0.048 (7)*	

### Atomic displacement parameters (Å^2^)

**Table:**

	*U*^11^	*U*^22^	*U*^33^	*U*^12^	*U*^13^	*U*^23^
Br1	0.01641 (7)	0.02614 (8)	0.02309 (7)	0.00403 (5)	0.00799 (5)	0.01061 (6)
Br2	0.01825 (7)	0.02841 (8)	0.02049 (7)	0.00428 (5)	0.00856 (5)	0.01277 (6)
N1	0.0142 (5)	0.0200 (5)	0.0152 (5)	0.0023 (4)	0.0050 (4)	0.0069 (4)
N2	0.0143 (5)	0.0208 (6)	0.0172 (5)	0.0032 (4)	0.0067 (4)	0.0081 (5)
N3	0.0144 (5)	0.0243 (6)	0.0144 (5)	0.0029 (4)	0.0046 (4)	0.0087 (5)
N4	0.0181 (5)	0.0254 (6)	0.0185 (5)	0.0043 (5)	0.0083 (4)	0.0108 (5)
C1	0.0166 (6)	0.0182 (6)	0.0158 (6)	0.0028 (5)	0.0064 (5)	0.0064 (5)
C2	0.0159 (6)	0.0175 (6)	0.0166 (6)	0.0039 (5)	0.0077 (5)	0.0063 (5)
C3	0.0211 (6)	0.0203 (6)	0.0169 (6)	0.0055 (5)	0.0074 (5)	0.0078 (5)
C4	0.0262 (7)	0.0201 (6)	0.0186 (6)	0.0053 (5)	0.0112 (6)	0.0089 (5)
C5	0.0215 (7)	0.0210 (7)	0.0218 (7)	0.0023 (5)	0.0119 (6)	0.0068 (6)
C6	0.0157 (6)	0.0205 (6)	0.0202 (6)	0.0024 (5)	0.0079 (5)	0.0067 (5)
C7	0.0163 (6)	0.0171 (6)	0.0152 (6)	0.0026 (5)	0.0059 (5)	0.0060 (5)
C8	0.0159 (6)	0.0211 (6)	0.0155 (6)	0.0017 (5)	0.0025 (5)	0.0066 (5)
C9	0.0164 (6)	0.0214 (6)	0.0161 (6)	0.0029 (5)	0.0061 (5)	0.0087 (5)
C10	0.0194 (6)	0.0242 (7)	0.0199 (7)	0.0030 (5)	0.0041 (5)	0.0090 (6)
C11	0.0299 (8)	0.0224 (7)	0.0204 (7)	0.0030 (6)	0.0084 (6)	0.0071 (6)
C12	0.0350 (8)	0.0250 (7)	0.0243 (7)	0.0127 (6)	0.0144 (7)	0.0118 (6)
C13	0.0251 (7)	0.0347 (8)	0.0228 (7)	0.0131 (6)	0.0116 (6)	0.0163 (6)
C14	0.0169 (6)	0.0276 (7)	0.0159 (6)	0.0048 (5)	0.0071 (5)	0.0110 (6)
C15	0.0145 (6)	0.0327 (8)	0.0153 (6)	0.0032 (5)	0.0050 (5)	0.0119 (6)
C16	0.0172 (6)	0.0221 (6)	0.0152 (6)	0.0038 (5)	0.0042 (5)	0.0088 (5)
C17	0.0172 (6)	0.0272 (7)	0.0202 (7)	0.0042 (5)	0.0056 (5)	0.0111 (6)
C18	0.0193 (7)	0.0274 (7)	0.0235 (7)	0.0030 (6)	0.0022 (6)	0.0100 (6)
C19	0.0259 (7)	0.0301 (8)	0.0162 (6)	0.0045 (6)	0.0017 (6)	0.0080 (6)
C20	0.0267 (7)	0.0294 (8)	0.0173 (6)	0.0063 (6)	0.0078 (6)	0.0097 (6)
C21	0.0188 (6)	0.0229 (7)	0.0181 (6)	0.0044 (5)	0.0062 (5)	0.0096 (5)
C22	0.0162 (6)	0.0241 (7)	0.0180 (6)	0.0038 (5)	0.0055 (5)	0.0107 (5)
C23	0.0144 (6)	0.0255 (7)	0.0204 (6)	0.0062 (5)	0.0073 (5)	0.0097 (6)
C24	0.0170 (6)	0.0262 (7)	0.0201 (7)	0.0032 (5)	0.0053 (5)	0.0093 (6)
C25	0.0299 (8)	0.0288 (8)	0.0273 (8)	0.0018 (6)	0.0098 (6)	0.0146 (7)
C26	0.0244 (7)	0.0263 (8)	0.0414 (9)	0.0019 (6)	0.0123 (7)	0.0133 (7)
C27	0.0341 (9)	0.0273 (8)	0.0314 (8)	0.0028 (7)	0.0096 (7)	0.0117 (7)
C28	0.0601 (10)	0.0289 (7)	0.0402 (8)	0.0077 (6)	0.0086 (7)	0.0084 (6)
C29	0.0601 (10)	0.0289 (7)	0.0402 (8)	0.0077 (6)	0.0086 (7)	0.0084 (6)
C30	0.0207 (7)	0.0316 (8)	0.0224 (7)	0.0046 (6)	0.0120 (6)	0.0125 (6)
C31	0.021 (2)	0.025 (3)	0.026 (3)	0.003 (2)	0.010 (2)	0.011 (2)
C32	0.026 (3)	0.0297 (9)	0.012 (3)	−0.0043 (14)	0.004 (2)	0.0073 (17)
C33	0.037 (2)	0.0225 (19)	0.030 (2)	0.0061 (16)	0.0182 (17)	0.0121 (17)
C34	0.032 (3)	0.027 (2)	0.023 (3)	0.005 (2)	0.009 (2)	0.0084 (18)
C35	0.0307 (18)	0.033 (2)	0.030 (2)	0.0012 (15)	0.0108 (15)	0.0166 (18)
C36	0.038 (3)	0.062 (3)	0.042 (3)	0.003 (3)	0.017 (3)	0.030 (2)
C31X	0.031 (3)	0.030 (3)	0.037 (3)	0.008 (2)	0.017 (3)	0.021 (2)
C32X	0.026 (3)	0.0297 (9)	0.012 (3)	−0.0043 (14)	0.004 (2)	0.0073 (17)
C33X	0.0217 (14)	0.0299 (19)	0.0300 (18)	0.0033 (13)	0.0101 (13)	0.0146 (16)
C34X	0.023 (2)	0.0247 (18)	0.024 (2)	0.0026 (18)	0.0094 (19)	0.0138 (16)
C35X	0.0295 (16)	0.0322 (19)	0.0263 (17)	0.0002 (13)	0.0099 (13)	0.0135 (16)
C36X	0.035 (2)	0.042 (2)	0.039 (2)	0.002 (2)	0.017 (2)	0.0231 (19)
O1W	0.0238 (6)	0.0322 (7)	0.0347 (7)	−0.0010 (5)	−0.0019 (5)	0.0163 (6)

### Geometric parameters (Å, °)

**Table:**

N1—C1	1.3391 (17)	C25—H25B	0.9900
N1—C7	1.3961 (17)	C26—C27	1.517 (2)
N1—C8	1.4618 (18)	C26—H26A	0.9900
N2—C1	1.3304 (18)	C26—H26B	0.9900
N2—C2	1.3965 (17)	C27—C28	1.520 (3)
N2—C23	1.4799 (18)	C27—H27A	0.9900
N3—C22	1.3348 (17)	C27—H27B	0.9900
N3—C16	1.3928 (17)	C28—C29	1.527 (3)
N3—C15	1.4808 (18)	C28—H28A	0.9900
N4—C22	1.3377 (18)	C28—H28B	0.9900
N4—C21	1.3940 (18)	C29—H29A	0.9800
N4—C30	1.4740 (18)	C29—H29B	0.9800
C1—H1A	0.9500	C29—H29C	0.9800
C2—C7	1.3940 (19)	C30—C31	1.475 (8)
C2—C3	1.3949 (19)	C30—C31X	1.580 (7)
C3—C4	1.389 (2)	C30—H30A	0.9900
C3—H3A	0.9500	C30—H30B	0.9900
C4—C5	1.408 (2)	C30—H30C	0.9600
C4—H4A	0.9500	C30—H30D	0.9600
C5—C6	1.386 (2)	C31—C32	1.496 (9)
C5—H5A	0.9500	C31—H31A	0.9900
C6—C7	1.3947 (19)	C31—H31D	0.9900
C6—H6A	0.9500	C32—C33	1.560 (10)
C8—C9	1.519 (2)	C32—H32A	0.9900
C8—H8A	0.9900	C32—H32D	0.9900
C8—H8B	0.9900	C33—C34	1.529 (7)
C9—C10	1.3955 (19)	C33—H33A	0.9900
C9—C14	1.404 (2)	C33—H33B	0.9900
C10—C11	1.392 (2)	C34—C35	1.511 (7)
C10—H10A	0.9500	C34—H34A	0.9900
C11—C12	1.386 (2)	C34—H34B	0.9900
C11—H11A	0.9500	C35—C36	1.508 (6)
C12—C13	1.386 (2)	C35—H35A	0.9900
C12—H12A	0.9500	C35—H35B	0.9900
C13—C14	1.398 (2)	C36—H36A	0.9800
C13—H13A	0.9500	C36—H36B	0.9800
C14—C15	1.507 (2)	C36—H36C	0.9800
C15—H15A	0.9900	C31X—C32X	1.534 (9)
C15—H15B	0.9900	C31X—H31B	0.9900
C16—C21	1.392 (2)	C31X—H31C	0.9900
C16—C17	1.3936 (19)	C32X—C33X	1.536 (8)
C17—C18	1.387 (2)	C32X—H32B	0.9900
C17—H17A	0.9500	C32X—H32C	0.9900
C18—C19	1.404 (2)	C33X—C34X	1.522 (6)
C18—H18A	0.9500	C33X—H33C	0.9900
C19—C20	1.384 (2)	C33X—H33D	0.9900
C19—H19A	0.9500	C34X—C35X	1.529 (6)
C20—C21	1.394 (2)	C34X—H34C	0.9900
C20—H20A	0.9500	C34X—H34D	0.9900
C22—H22A	0.9500	C35X—C36X	1.524 (5)
C23—C24	1.518 (2)	C35X—H35C	0.9900
C23—H23A	0.9900	C35X—H35D	0.9900
C23—H23B	0.9900	C36X—H36D	0.9800
C24—C25	1.522 (2)	C36X—H36E	0.9800
C24—H24A	0.9900	C36X—H36F	0.9800
C24—H24B	0.9900	O1W—H1W1	0.84 (2)
C25—C26	1.533 (2)	O1W—H2W1	0.79 (3)
C25—H25A	0.9900		
			
C1—N1—C7	108.14 (11)	C27—C26—H26B	108.8
C1—N1—C8	125.77 (12)	C25—C26—H26B	108.8
C7—N1—C8	126.08 (11)	H26A—C26—H26B	107.7
C1—N2—C2	108.59 (11)	C26—C27—C28	114.01 (16)
C1—N2—C23	125.85 (12)	C26—C27—H27A	108.8
C2—N2—C23	125.50 (12)	C28—C27—H27A	108.8
C22—N3—C16	108.14 (11)	C26—C27—H27B	108.8
C22—N3—C15	128.98 (12)	C28—C27—H27B	108.8
C16—N3—C15	122.61 (11)	H27A—C27—H27B	107.6
C22—N4—C21	108.35 (12)	C27—C28—C29	112.23 (19)
C22—N4—C30	125.98 (12)	C27—C28—H28A	109.2
C21—N4—C30	125.65 (12)	C29—C28—H28A	109.2
N2—C1—N1	110.19 (12)	C27—C28—H28B	109.2
N2—C1—H1A	124.9	C29—C28—H28B	109.2
N1—C1—H1A	124.9	H28A—C28—H28B	107.9
C7—C2—C3	122.01 (13)	C28—C29—H29A	109.5
C7—C2—N2	106.38 (12)	C28—C29—H29B	109.5
C3—C2—N2	131.57 (13)	H29A—C29—H29B	109.5
C4—C3—C2	115.83 (13)	C28—C29—H29C	109.5
C4—C3—H3A	122.1	H29A—C29—H29C	109.5
C2—C3—H3A	122.1	H29B—C29—H29C	109.5
C3—C4—C5	122.12 (13)	N4—C30—C31	113.6 (3)
C3—C4—H4A	118.9	N4—C30—C31X	110.5 (3)
C5—C4—H4A	118.9	N4—C30—H30A	108.9
C6—C5—C4	121.84 (13)	C31—C30—H30A	108.9
C6—C5—H5A	119.1	N4—C30—H30B	108.9
C4—C5—H5A	119.1	C31—C30—H30B	108.9
C5—C6—C7	115.94 (13)	H30A—C30—H30B	107.7
C5—C6—H6A	122.0	N4—C30—H30C	110.1
C7—C6—H6A	122.0	C31—C30—H30C	93.8
C2—C7—C6	122.25 (13)	C31X—C30—H30C	110.5
C2—C7—N1	106.71 (11)	H30B—C30—H30C	121.1
C6—C7—N1	130.99 (13)	N4—C30—H30D	109.5
N1—C8—C9	112.83 (11)	C31—C30—H30D	120.1
N1—C8—H8A	109.0	C31X—C30—H30D	107.8
C9—C8—H8A	109.0	H30A—C30—H30D	93.7
N1—C8—H8B	109.0	H30C—C30—H30D	108.3
C9—C8—H8B	109.0	C30—C31—C32	119.6 (6)
H8A—C8—H8B	107.8	C30—C31—H31A	107.4
C10—C9—C14	118.88 (13)	C32—C31—H31A	107.4
C10—C9—C8	120.99 (13)	C30—C31—H31D	107.4
C14—C9—C8	120.13 (12)	C32—C31—H31D	107.4
C11—C10—C9	121.08 (14)	H31A—C31—H31D	106.9
C11—C10—H10A	119.5	C31—C32—C33	119.7 (9)
C9—C10—H10A	119.5	C31—C32—H32A	107.4
C12—C11—C10	119.96 (14)	C33—C32—H32A	107.4
C12—C11—H11A	120.0	C31—C32—H32D	107.4
C10—C11—H11A	120.0	C33—C32—H32D	107.4
C11—C12—C13	119.53 (15)	H32A—C32—H32D	106.9
C11—C12—H12A	120.2	C34—C33—C32	110.0 (6)
C13—C12—H12A	120.2	C34—C33—H33A	109.7
C12—C13—C14	121.15 (14)	C32—C33—H33A	109.7
C12—C13—H13A	119.4	C34—C33—H33B	109.7
C14—C13—H13A	119.4	C32—C33—H33B	109.7
C13—C14—C9	119.41 (13)	H33A—C33—H33B	108.2
C13—C14—C15	117.53 (13)	C35—C34—C33	114.8 (5)
C9—C14—C15	122.95 (13)	C35—C34—H34A	108.6
N3—C15—C14	114.66 (11)	C33—C34—H34A	108.6
N3—C15—H15A	108.6	C35—C34—H34B	108.6
C14—C15—H15A	108.6	C33—C34—H34B	108.6
N3—C15—H15B	108.6	H34A—C34—H34B	107.5
C14—C15—H15B	108.6	C36—C35—C34	112.6 (5)
H15A—C15—H15B	107.6	C36—C35—H35A	109.1
C21—C16—N3	106.94 (12)	C34—C35—H35A	109.1
C21—C16—C17	122.16 (13)	C36—C35—H35B	109.1
N3—C16—C17	130.88 (13)	C34—C35—H35B	109.1
C18—C17—C16	115.95 (14)	H35A—C35—H35B	107.8
C18—C17—H17A	122.0	C32X—C31X—C30	104.7 (5)
C16—C17—H17A	122.0	C32X—C31X—H31B	110.8
C17—C18—C19	121.77 (14)	C30—C31X—H31B	110.8
C17—C18—H18A	119.1	C32X—C31X—H31C	110.8
C19—C18—H18A	119.1	C30—C31X—H31C	110.8
C20—C19—C18	122.23 (14)	H31B—C31X—H31C	108.9
C20—C19—H19A	118.9	C31X—C32X—C33X	114.6 (7)
C18—C19—H19A	118.9	C31X—C32X—H32B	108.6
C19—C20—C21	115.87 (14)	C33X—C32X—H32B	108.6
C19—C20—H20A	122.1	C31X—C32X—H32C	108.6
C21—C20—H20A	122.1	C33X—C32X—H32C	108.6
C16—C21—C20	122.01 (13)	H32B—C32X—H32C	107.6
C16—C21—N4	106.38 (12)	C34X—C33X—C32X	113.1 (5)
C20—C21—N4	131.60 (14)	C34X—C33X—H33C	109.0
N3—C22—N4	110.19 (12)	C32X—C33X—H33C	109.0
N3—C22—H22A	124.9	C34X—C33X—H33D	109.0
N4—C22—H22A	124.9	C32X—C33X—H33D	109.0
N2—C23—C24	112.21 (12)	H33C—C33X—H33D	107.8
N2—C23—H23A	109.2	C33X—C34X—C35X	113.3 (4)
C24—C23—H23A	109.2	C33X—C34X—H34C	108.9
N2—C23—H23B	109.2	C35X—C34X—H34C	108.9
C24—C23—H23B	109.2	C33X—C34X—H34D	108.9
H23A—C23—H23B	107.9	C35X—C34X—H34D	108.9
C23—C24—C25	113.95 (13)	H34C—C34X—H34D	107.7
C23—C24—H24A	108.8	C36X—C35X—C34X	113.0 (4)
C25—C24—H24A	108.8	C36X—C35X—H35C	109.0
C23—C24—H24B	108.8	C34X—C35X—H35C	109.0
C25—C24—H24B	108.8	C36X—C35X—H35D	109.0
H24A—C24—H24B	107.7	C34X—C35X—H35D	109.0
C24—C25—C26	112.82 (14)	H35C—C35X—H35D	107.8
C24—C25—H25A	109.0	C35X—C36X—H36D	109.5
C26—C25—H25A	109.0	C35X—C36X—H36E	109.5
C24—C25—H25B	109.0	H36D—C36X—H36E	109.5
C26—C25—H25B	109.0	C35X—C36X—H36F	109.5
H25A—C25—H25B	107.8	H36D—C36X—H36F	109.5
C27—C26—C25	113.75 (14)	H36E—C36X—H36F	109.5
C27—C26—H26A	108.8	H1W1—O1W—H2W1	106 (2)
C25—C26—H26A	108.8		
			
C2—N2—C1—N1	0.07 (16)	C22—N3—C16—C17	−178.32 (16)
C23—N2—C1—N1	177.39 (12)	C15—N3—C16—C17	7.2 (2)
C7—N1—C1—N2	−0.09 (16)	C21—C16—C17—C18	−0.2 (2)
C8—N1—C1—N2	179.03 (12)	N3—C16—C17—C18	178.08 (15)
C1—N2—C2—C7	−0.02 (15)	C16—C17—C18—C19	0.5 (2)
C23—N2—C2—C7	−177.35 (12)	C17—C18—C19—C20	−0.4 (3)
C1—N2—C2—C3	177.66 (15)	C18—C19—C20—C21	0.0 (2)
C23—N2—C2—C3	0.3 (2)	N3—C16—C21—C20	−178.90 (14)
C7—C2—C3—C4	0.0 (2)	C17—C16—C21—C20	−0.3 (2)
N2—C2—C3—C4	−177.37 (14)	N3—C16—C21—N4	0.06 (16)
C2—C3—C4—C5	0.2 (2)	C17—C16—C21—N4	178.71 (14)
C3—C4—C5—C6	−0.3 (2)	C19—C20—C21—C16	0.4 (2)
C4—C5—C6—C7	0.1 (2)	C19—C20—C21—N4	−178.30 (15)
C3—C2—C7—C6	−0.2 (2)	C22—N4—C21—C16	−0.27 (16)
N2—C2—C7—C6	177.79 (13)	C30—N4—C21—C16	−178.69 (13)
C3—C2—C7—N1	−177.98 (12)	C22—N4—C21—C20	178.56 (16)
N2—C2—C7—N1	−0.03 (15)	C30—N4—C21—C20	0.1 (3)
C5—C6—C7—C2	0.1 (2)	C16—N3—C22—N4	−0.34 (17)
C5—C6—C7—N1	177.34 (14)	C15—N3—C22—N4	173.71 (14)
C1—N1—C7—C2	0.07 (15)	C21—N4—C22—N3	0.38 (17)
C8—N1—C7—C2	−179.04 (12)	C30—N4—C22—N3	178.80 (13)
C1—N1—C7—C6	−177.48 (14)	C1—N2—C23—C24	100.16 (16)
C8—N1—C7—C6	3.4 (2)	C2—N2—C23—C24	−82.96 (17)
C1—N1—C8—C9	−101.05 (16)	N2—C23—C24—C25	−73.02 (16)
C7—N1—C8—C9	77.90 (16)	C23—C24—C25—C26	−175.79 (13)
N1—C8—C9—C10	15.32 (18)	C24—C25—C26—C27	−67.89 (19)
N1—C8—C9—C14	−165.31 (12)	C25—C26—C27—C28	−177.48 (16)
C14—C9—C10—C11	0.2 (2)	C26—C27—C28—C29	−177.98 (17)
C8—C9—C10—C11	179.59 (13)	C22—N4—C30—C31	−113.8 (3)
C9—C10—C11—C12	−0.3 (2)	C21—N4—C30—C31	64.4 (3)
C10—C11—C12—C13	0.3 (2)	C22—N4—C30—C31X	−95.1 (3)
C11—C12—C13—C14	−0.1 (2)	C21—N4—C30—C31X	83.1 (3)
C12—C13—C14—C9	−0.1 (2)	N4—C30—C31—C32	176.3 (6)
C12—C13—C14—C15	176.19 (14)	C31X—C30—C31—C32	92.5 (18)
C10—C9—C14—C13	0.0 (2)	C30—C31—C32—C33	−147.7 (6)
C8—C9—C14—C13	−179.39 (13)	C31—C32—C33—C34	−157.1 (7)
C10—C9—C14—C15	−176.05 (13)	C32—C33—C34—C35	−170.0 (5)
C8—C9—C14—C15	4.6 (2)	C33—C34—C35—C36	−170.2 (4)
C22—N3—C15—C14	25.2 (2)	N4—C30—C31X—C32X	−174.1 (4)
C16—N3—C15—C14	−161.53 (13)	C31—C30—C31X—C32X	−70.8 (16)
C13—C14—C15—N3	97.87 (16)	C30—C31X—C32X—C33X	154.5 (6)
C9—C14—C15—N3	−86.02 (17)	C31X—C32X—C33X—C34X	154.0 (6)
C22—N3—C16—C21	0.16 (16)	C32X—C33X—C34X—C35X	175.9 (6)
C15—N3—C16—C21	−174.34 (13)	C33X—C34X—C35X—C36X	179.2 (4)

### Hydrogen-bond geometry (Å, °)

**Table:**

*D*—H···*A*	*D*—H	H···*A*	*D*···*A*	*D*—H···*A*
O1W—H1W1···Br1	0.84 (3)	2.50 (3)	3.3271 (17)	169 (2)
O1W—H2W1···Br2	0.79 (3)	2.54 (3)	3.3280 (14)	177 (3)
C1—H1A···Br1^i^	0.95	2.80	3.6093 (15)	144
C3—H3A···Br2^ii^	0.95	2.92	3.7866 (16)	153
C5—H5A···Br2^iii^	0.95	2.89	3.8162 (17)	167
C8—H8A···Br2^iv^	0.99	2.93	3.9117 (16)	172
C15—H15A···Br2^iv^	0.99	2.72	3.6809 (19)	165
C15—H15B···Br1^iv^	0.99	2.80	3.7842 (15)	170
C18—H18A···O1W^v^	0.95	2.46	3.187 (2)	133
C20—H20A···Br2	0.95	2.76	3.6602 (16)	158
C22—H22A···Br1^i^	0.95	2.70	3.5577 (15)	150
C23—H23A···Br2^i^	0.99	2.89	3.7836 (14)	151
C23—H23B···Br2^ii^	0.99	2.81	3.7285 (17)	154

Symmetry codes: (i) −*x*+1, −*y*+1, −*z*+1; (ii) *x*+1, *y*, *z*+1; (iii) *x*, *y*, *z*+1; (iv) −*x*, −*y*+1, −*z*+1; (v) *x*−1, *y*, *z*.
